# Role of plasma metabolome in mediating the effect of plasma lipidome on NAFLD: a Mendelian randomization study

**DOI:** 10.3389/fendo.2024.1436827

**Published:** 2025-01-23

**Authors:** Zhuyuan Zheng, Shaojie Yang, Wanlin Dai, Yang Sun, Jingnan Wang, Xiaolin Zhang, Yiming Zheng, Jing Kong

**Affiliations:** ^1^ Biliary Surgery (2nd General) Unit, Department of General Surgery, Shengjing Hospital of China Medical University, Shenyang, Liaoning, China; ^2^ Innovation Institute of China Medical University, Shenyang, Liaoning, China

**Keywords:** Mendelian randomization (MR), nonalcoholic fatty liver disease (NAFLD), plasma lipidome, plasma metabolome, mediation analysis

## Abstract

**Background:**

This study explored the causal connection among the plasma lipidome, nonalcoholic fatty liver disease (NAFLD), and potential metabolome mediators through Mendelian randomization (MR).

**Methods:**

We obtained summary statistics for 179 plasma lipidome traits (*N* = 7,174), 1,400 plasma metabolome traits (*N* = 8,299), and one NAFLD trait from publicly available genome-wide association studies. A two-sample MR analysis was conducted to infer causality. Additionally, multiple sensitivity analyses were conducted to assess the heterogeneity, horizontal pleiotropy, and robustness of the MR findings. MetaboAnalyst 6.0 was used for the pathway analysis of the identified lipids and metabolites. Furthermore, we used mediation analysis to assess whether the effect of plasma lipidome on NAFLD was mediated by plasma metabolome.

**Results:**

The MR analysis predicted a genetically determined causal relationship between plasma lipidomes and NAFLD. No compelling proof was found that genetically predicted NAFLD influenced the risk of the five plasma lipidomes mentioned earlier. Based on established causal relationships between lipids and metabolites, we identified that eight metabolic pathways are closely associated with NAFLD. Our mediation analysis revealed six mediation relationships, indicating the causal pathway from plasma lipids to NAFLD mediated by five specific metabolites. No potential pleiotropy was found in the sensitivity analysis.

**Conclusions:**

In summary, our study identified causal relationships between plasma lipidomes, plasma metabolomes, and NAFLD. Certainly, the impact of plasma lipidomes on NAFLD is not limited to plasma metabolomes, indicating a need to further investigate into other possible mediators. These identified factors may become new biomarkers of the NAFLD contributing to its prevention, diagnosis, and treatment.

## 1 Introduction

Nonalcoholic fatty liver disease (NAFLD) is characterized as a chronic inflammatory disease of the liver with hepatic fat accumulation, mainly triacylglycerol (TAG). In NAFLD, fat accumulation progresses to nonalcoholic steatohepatitis (NASH), which can lead to cirrhosis and hepatocarcinoma (1). The majority of individuals with NAFLD do not experience any symptoms until they develop liver cirrhosis. NAFLD is becoming a worldwide public health epidemic, of which the prevalence ranges up to 25%, posing a substantial burden on medical, economic, and social aspects (2). NAFLD was defined as chronic liver disease with steatosis in more than 5% of hepatocytes mostly attributable to metabolic factors, without the induction of excess alcohol use and other clear evidence. To date, diagnosing the condition becomes notably more difficult in the absence of histological examination, and no effective treatment has been identified as well. Therefore, future studies on sensitive diagnostic biomarkers and highly effective therapeutic approaches for NAFLD are pivotal (3).

Dysfunction in lipid metabolism acts as a pivotal hub of the pathogenesis of liver metabolic diseases, revealing that the plasma lipidome captures risk for NAFLD (4). In recent years, omics technology (genomics, metabolomics, lipidomics, etc.) has been widely used in NAFLD studies (5, 6). Lipidomics techniques enable large-scale and comprehensive studies of lipids in biological samples, which contribute to facilitate understanding of the alterations of lipid metabolism on a biological system. Several previous studies identified a list of NAFLD-related plasma lipid biomarkers that mainly belong to triacylglycerol, phospholipids, and sphingolipid classes (6). However, the key lipid biomarkers reported in multiple studies rarely overlapped, which limits the widespread clinical application. A previous Mendelian randomization (MR) study showed that causality could not be inferred between lipid traits (such as TAG, low-density lipoprotein cholesterol and total cholesterol) and NAFLD and that lipid-lowering medications exerted a beneficial impact (7). The opposite conclusion has been drawn from another MR study: that low-density lipoprotein cholesterol and TAG increased NAFLD risk (8). The similarity between fatty acids (FAs) from liver TAG pools and those used for TAG secretion through lipoproteins (9) indicates that serum lipid profiles may reflect liver lipid composition (10). Therefore, additional research are necessary to explore the plasma lipidome in relation to NAFLD due to the current lack of conclusive evidence.

Multiple research studies have indicated a strong connection between NAFLD and metabolic disorders (8), with insulin resistance being identified as a key element (11). Metabolomics aims to uncover changes in metabolites and metabolic pathways of tissues under different physiological or pathological states. Metabolome has the potential to provide useful information for the prediction of metabolic phenotypes. Compared with lipidomics focusing entirely on the measurement of lipid and lipid-like molecules, metabolomics emphasizes polar or aqueously soluble metabolites, such as sugars, amino acids, and nucleotides (12). Prior studies have suggested that specific metabolomics signatures were associated to different stages of NAFLD (6). Potential pathways related to plasma lipids and metabolites on NAFLD have not been investigated. With a high degree of interconnectivity between lipid metabolism and other metabolic pathways for maintaining lipid homeostasis, the combination of metabolomes and lipidomes can fully exploit their role in comprehending the molecular processes underlying diseases (6, 12).

Although previous studies have identified the relationship between plasma lipidomes, metabolomes, and NAFLD, the exact causal connections and their respective mediation proportions remain unclear. With the extensive usage of high-throughput technologies in NAFLD research, there has been a rapid growth in the amount of publicly available datasets. Utilizing the genetic variants as instrumental variables (IVs) and large-scale data from genome-wide association studies (GWASs), MR has become a popular epidemiological method that focuses on identifying potential causal relationships between exposures and outcomes (13). Inherited genetic variants are randomly assigned at conception and unalterable by nonmeasurement errors, confounding factors, or reverse causality, which enhances the accuracy and objectivity of MR analysis (13). Additionally, mediation analysis dissects the overall causal effect of exposures on outcomes by examining the indirect impact of exposures on outcomes through mediation factors (14). We conducted MR analyses using publicly available GWAS summary data to investigate the potential causal relationships between plasma lipids, plasma metabolites, and NAFLD as well as to identify pathways connecting plasma lipids to NAFLD mediated by plasma metabolites.

## 2 Methods

### 2.1 Study design

**Figure 1:**
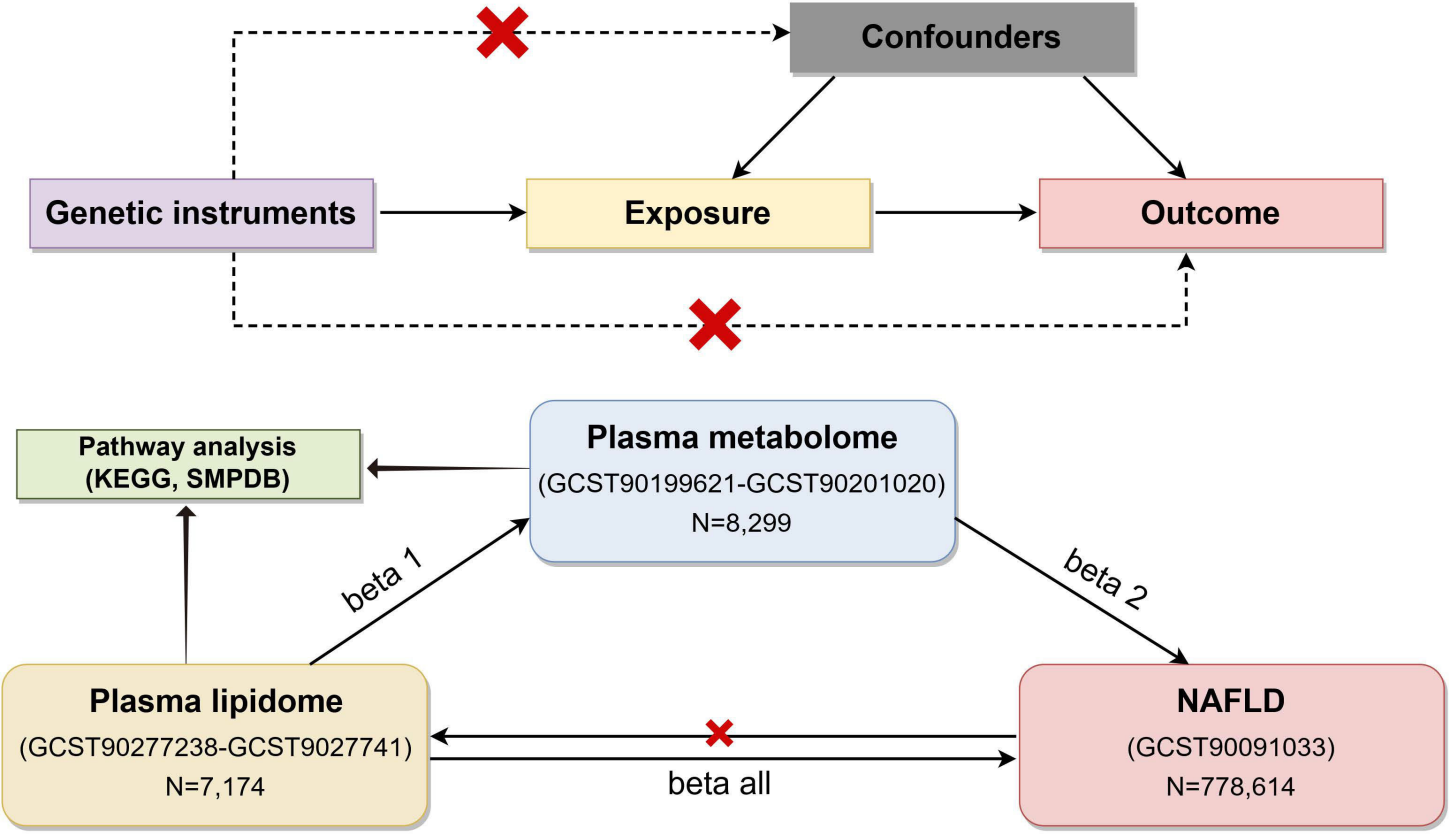
Flow chart of the study (by Figdraw). To estimate causal effects using genetic variation in a MR study, three key assumptions must be satisfied: (1) the IVs are associated with the exposure, (2) the IVs influence the outcome exclusively through the exposure, and (3) the IVs are not correlated with any confounders. MR, Mendelian randomization; NAFLD, nonalcoholic fatty liver disease; IVs, instrumental variables.

**Figure 1** illustrates the flowchart of the study. Firstly, we obtained summary statistics from published GWAS data, including plasma lipidome, plasma metabolome, and NAFLD traits. Next, we explored the causal relationship between plasma lipids and NAFLD using bidirectional two-sample MR analyses. Lastly, a two-step analysis was performed to investigate how plasma metabolites mediate the connection between plasma lipids and NAFLD. The data used in our analysis were approved by their respective institutional review committees. This study followed the STROBE-MR guidelines (15). In our study, single-nucleotide polymorphisms (SNPs) were utilized as IVs.

### 2.2 Data sources

The study used publicly available data from participants of European descent. Complete summary statistics for the plasma lipidomes can be found in the GWAS catalog (https://www.ebi.ac.uk/gwas/) with the registry number GCST90277238–GCST9027741, which included 179 plasma lipids from 7,174 European individuals from the prospective GeneRISK cohort (16). In this study, 179 plasma lipids can be divided into four categories and 13 lipid classes, including phosphatidylinositol (10), phosphatidylethanolamine-ether (18), phosphatidylethanolamine (5), phosphatidylcholine-ether (27), phosphatidylcholine (PC) (46), lysophosphatidylethanolamine (3), lysophosphatidylcholine (5), triacylglycerol (38), diacylglycerol (6), ceramide (4), sphingomyelin (11), cholesteryl ester (15), and cholesterol (1, 16).

The plasma metabolomics summary statistics for 8,299 European individuals can be found in the GWAS Catalog with the registry number GCST90199621–GCST90201020 (17). The dataset comprises 1,091 metabolites and 309 metabolite ratios (17).

The GWAS summary statistics for NAFLD under the registry number GCST90091033 were sourced from a meta-analysis that integrated data from four European GWAS studies, namely, the Electronic Medical Records and Genomics, UK Biobank, FinnGen, and Estonian Biobank (18). Data on the relationships of exposure-associated SNPs with NAFLD contained 8,434 NAFLD cases and 770,180 non-cases (18). Quality control details are provided in the referenced GWAS publications (16–18).

### 2.3 Instrumental variable selection

To accurately estimate causal effects using genetic variation, it is crucial that the IVs influence the outcome solely through the exposure. The specific screening conditions are as follows. In cases when SNPs did not have a significant genome-wide impact, SNPs with a lower significance level (*P*< 1×10^-5^) were used as IVs in plasma lipidome and metabolome GWASs (19). SNPs with higher significance (*P*< 5×10^-8^) were included in NAFLD GWAS (19, 20). Clumping was employed on SNPs according to linkage disequilibrium (*r* ^2^< 0.001, window size = 10,000 kb).


$$\text{F}=\frac{\left(\text{N}-2\right)\times {\text{R}}^{2}}{\left(1-{\text{R}}^{2}\right)}$$

*N* denotes the sample size, while *R*² represents the amount of variance in the exposure that is explained by the IVs. *F*‐statistic >10 was used as a screening standard in the selection of strong IVs (21). To exclude SNPs with an invalid causal direction, MR Steiger filtering was employed.

### 2.4 Statistical analysis

All MR analyses in our study were conducted in R software (version 4.3.3), utilizing the package “TwoSampleMR” (version 0.5.10) (22).

#### 2.4.1 Two-sample Mendelian randomization

The MR approach was employed to assess the causal relationships between plasma lipid profiles, metabolomes, and NAFLD. The inverse variance weighted (IVW) method combines the Wald ratio of causal effects for each IVs in a meta-analysis, which shows the highest statistical power under the absence of horizontal pleiotropy. Other methods, including MR-Egger, weighted median, simple mode, and weighted mode, were used as supplements (13).

Sensitivity analyses were conducted to assess the robustness of causality. Horizontal pleiotropy arises when a single IV influences the outcome through multiple biological pathways, beyond its association with the exposure factor, thereby violating MR fundamental assumptions. The MR-Egger regression (23) and MR-PRESSO global test (24) can be used to detect the horizontal pleiotropy. The strict selection criterion of *P* > 0.05 was employed due to the non-zero MR-Egger regression intercept indicating directional pleiotropy. Heterogeneity refers to the variation in effect size and direction among IVs from different sources. To evaluate this heterogeneity, Cochran’s *Q* test was utilized, with a threshold of *P >*0.05 deemed enhanced. Even *P*<0.05 was acceptable for our analysis. The forest plot, scatter plot, and funnel plot were also generated to conduct a sensitivity analysis. Furthermore, leave-one-out analysis was utilized in our MR study to evaluate the impact of a specific SNP on the causal relationship. Only plasma lipidomes and metabolomes with no horizontal pleiotropy were considered in the subsequent analysis when the IVW MR method results were significant at *P*<0.05 for lipids and *P*<0.01 for metabolites.

#### 2.4.2 Reverse Mendelian randomization analysis

To investigate the potential causal relationship between NAFLD and the identified plasma lipids (*P*_IVW_< 0.05), a reverse MR analysis was conducted, in which NAFLD was treated as the exposure and plasma lipidomes were treated as the outcomes. The details of the reverse MR analysis procedure were similar to those in the MR analysis.

#### 2.4.3 Metabolic pathway analysis

MetaboAnalyst 6.0 (https://www.metaboanalyst.ca/) (25) was utilized to examine established plasma metabolites (*P*_IVW_< 0.05) in search of metabolic pathways associated with NAFLD using the KEGG database.

#### 2.4.4 Mediation analysis

Mediation analysis aims to assess how exposure affects the outcome through mediators, which is helpful to explore the underlying mechanism (14). This study aimed to assess and quantify the mediating role of plasma metabolomes on the association between plasma lipidomes and NAFLD. Firstly, a two-sample MR method was employed to assess the causal link of plasma lipids with metabolites, obtaining beta 1. Secondly, the causal relationship between plasma metabolites and NAFLD was evaluated with two-sample MR methods after adjusting for lipid-related SNPs to obtain beta 2. Lastly, beta all was calculated using the two-sample MR method to evaluate the causal link between plasma lipids and NAFLD. The mediation effect is calculated as beta 12 = beta 1 × beta 2 while the direct effect of plasma lipidomes on NAFLD as beta = beta all - beta 12. Thus, the proportion of the mediation effect is calculated using the following equation:


mediation proportion=(beta12/beta all) × 100%

The 95% confidence intervals were calculated using the delta method at the 95% confidence level (14). According to the findings, the identified metabolites were considered to exhibit potential mediation effects on the pathway linking the plasma lipids to NAFLD.

## 3 Results

### 3.1 Causal association of plasma lipidomes on NAFLD

**Figure 2:**
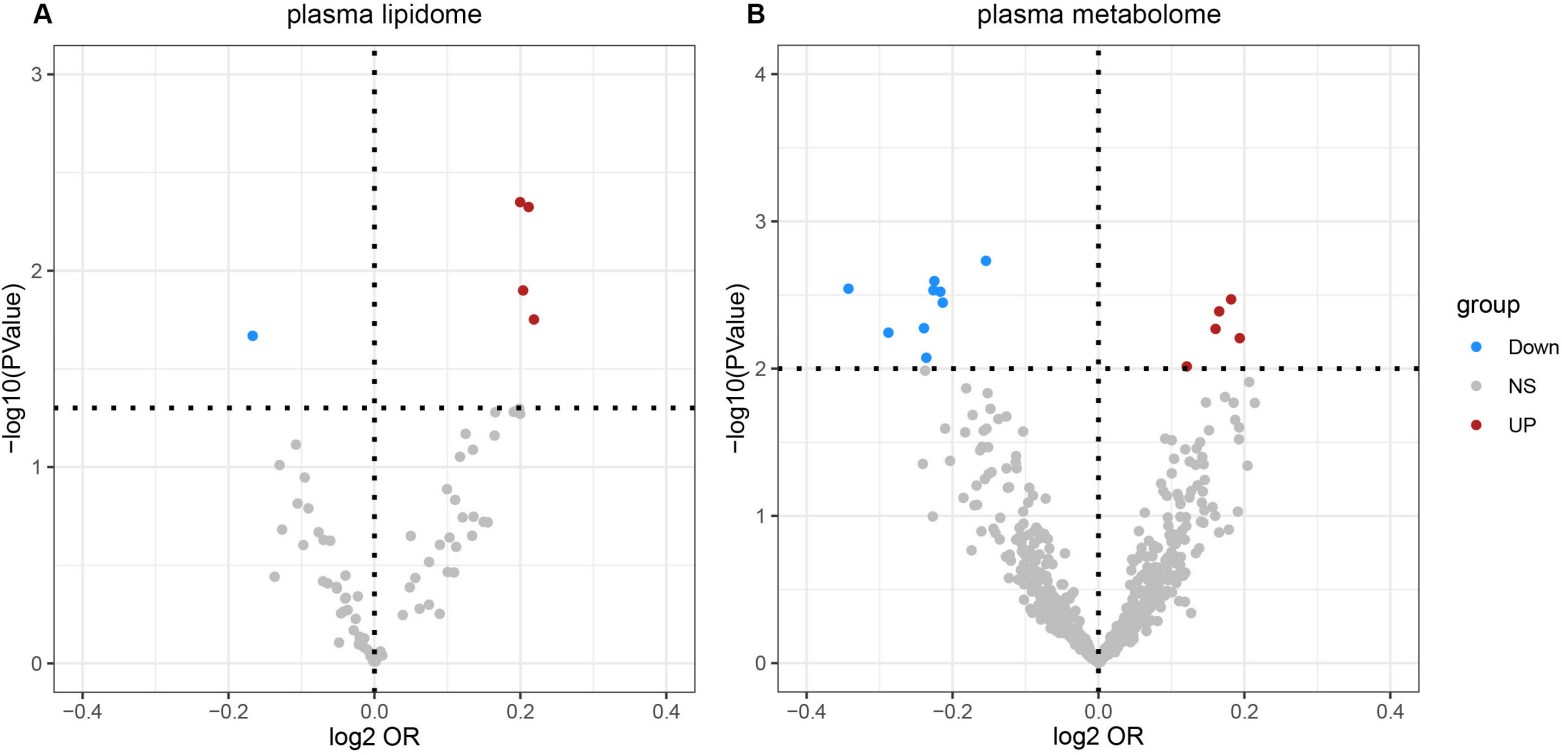
Volcano plots of the IVW MR for causal estimates of plasma lipidome on NAFLD. **(A)** Volcano plots of the IVW MR for causal estimates of plasma metabolome on NAFLD. **(B)** The causal estimates of the plasma lipidome on NAFLD were found to be statistically significant at the nominal threshold (*P*< 0.05). The causal estimates of the plasma metabolome on NAFLD were found to be statistically significant at the nominal threshold (*P*< 0.01). NAFLD, nonalcoholic fatty liver disease.

**Figure 3:**
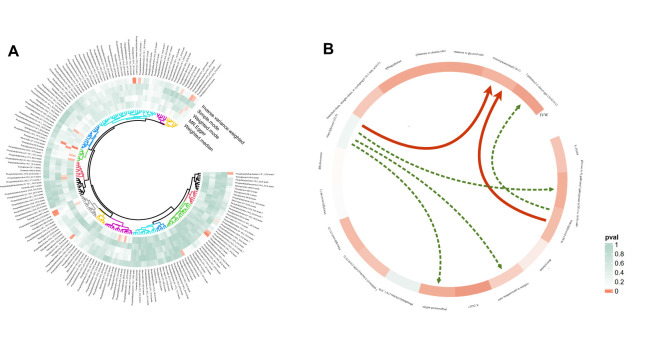
Circular heatmap of Mendelian randomization results of plasma lipidome and NAFLD **(A)** and the causal pathway from plasma lipidomes to NAFLD mediated by plasma metabolomes **(B)**. Six mediation relationships were identified, two of which exhibit statistical significance and four show potential implications. NAFLD, nonalcoholic fatty liver disease.

**Figure 4:**
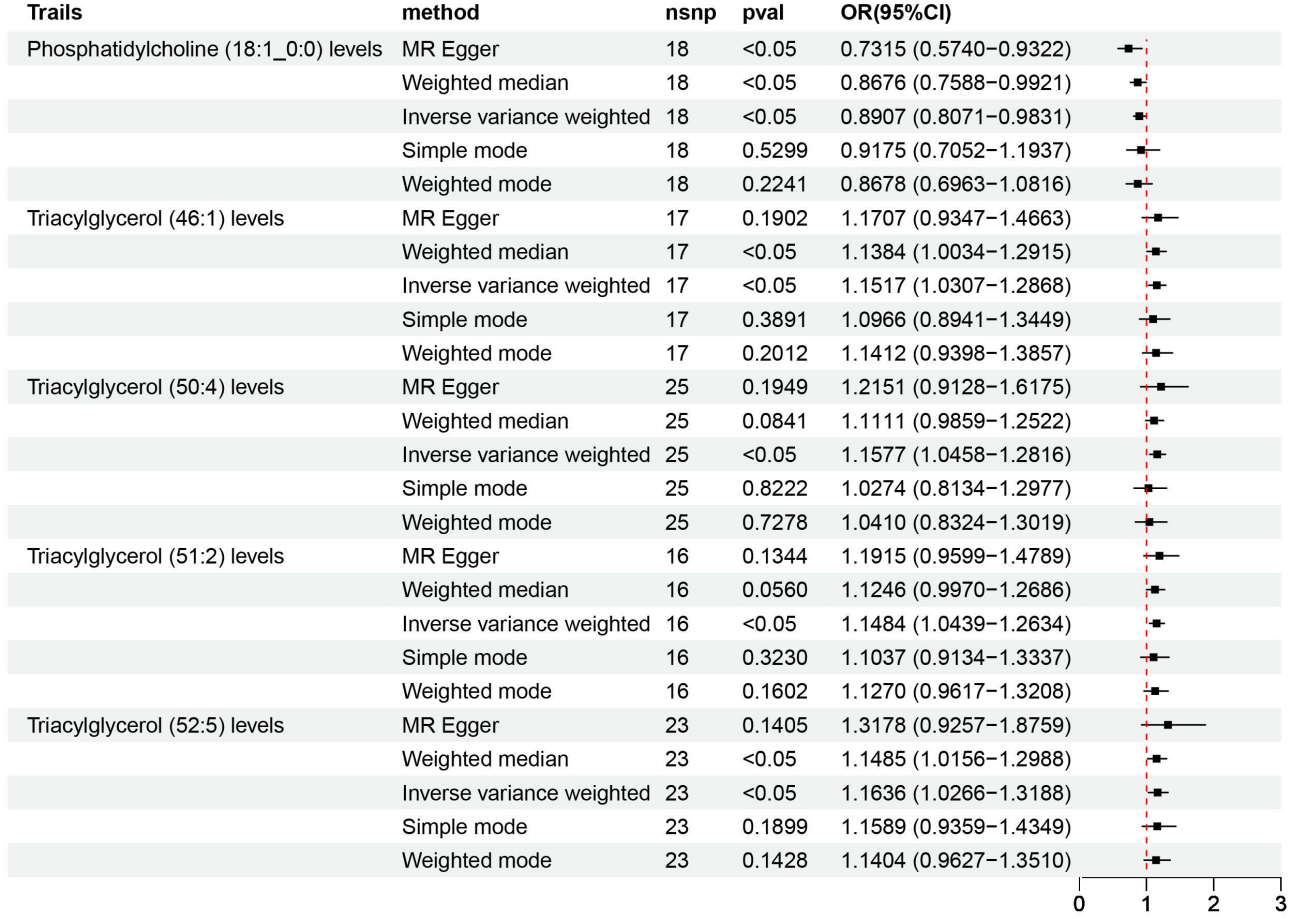
Causal estimates of MR between plasma lipidome and NAFLD. Estimates from MR analysis of plasma lipidome on NAFLD with five methods, namely, MR-Egger, weighted median, IVW, simple mode, and weighted mode. MR, Mendelian randomization; NAFLD, nonalcoholic fatty liver disease; IVW, inverse variance weighted.

**Figure 5:**
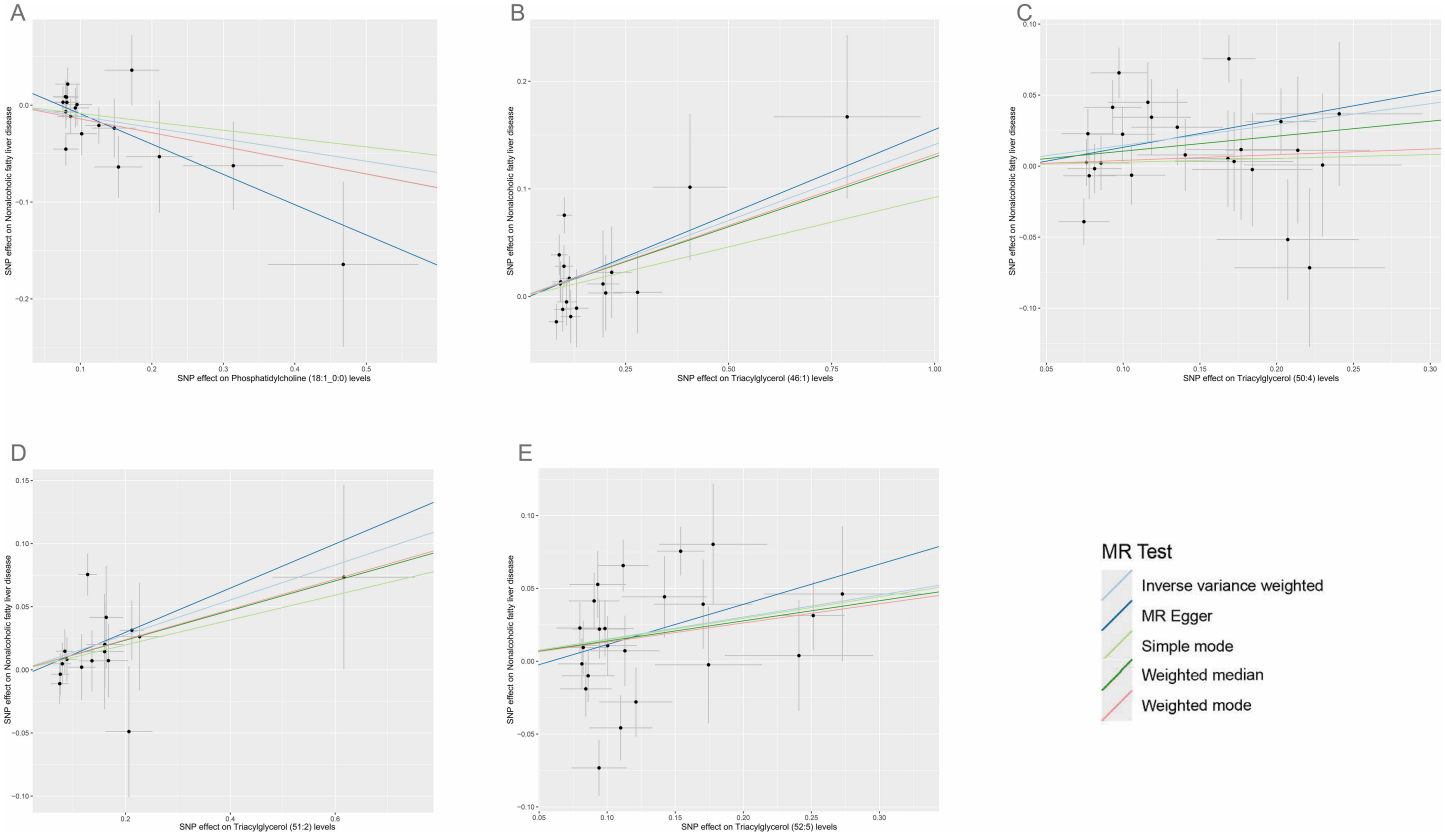
Scatter plots for the causal associations between five plasma lipid species on NAFLD. **(A–E)** Each black point represents the SNP effect sizes on the exposure (horizontal axis) and outcome (vertical axis) and is plotted with error bars of SE. The line slopes represent the causal association for each method: inverse variance weighted (light blue line), MR-Egger (blue line), simple mode (light green line), weighted median (green line), and weighted mode (red line). SNP, single-nucleotide polymorphisms; SE, standard error; IVW, inverse variance weighted.

According to IVW estimates, the five causal associations between genetically determined plasma lipidomes and NAFLD were demonstrated (*P*_IVW_< 0.05; corresponding to five plasma lipids) (**Figure 2A**). Among them, plasma lipidomes included TAG (4) and PC (1). Other methods, namely, MR-Egger, weighted median, simple mode, and weighted mode, were likewise utilized to enhance the results (**Figures 3A**–**5**; **Supplementary Table S2**). The identified IVs comprised 23, 25, 17, 16, and 18 SNPs, respectively, and were sufficiently effective for the MR analysis (*F*-statistic > 10). TAG (52:5) (primary MR analysis odds ratio (OR) 1.1636/SD increase, 95% confidence interval (CI) 1.0266−1.3188), TAG (50:4) (OR: 1.1577/SD increase, 95% CI: 1.0458−1.2816), TAG (46:1) (OR: 1.1517/SD increase, 95% CI: 1.0307−1.2868), and TAG (51:2) levels (OR: 1.1484/SD increase, 95% CI: 1.0439−1.2634) exhibited a positive causal relationship with NAFLD. In contrast, PC (18:1/0:0) levels (OR: 0.8907/SD increase, 95% CI: 0.8071−0.9831) had a significant negative causal relationship. This result is further visualized through the use of a scatter plot (**Figure 5**). The slope of the lines indicates the direction of causality, employing a color-coding scheme to differentiate among five MR analysis methods. The sensitivity analysis provided additional confirmation of the reliability of the results (**Supplementary Table S3**). The analysis indicated the absence of horizontal pleiotropy between five plasma lipids and NAFLD, as evidenced by a value of *P >*0.05 for the MR-Egger intercept, while mild-to-moderate heterogeneity existed. The MR-Steiger test showed no evidence of reverse causality. Alternatively, the leave-one-out sensitivity analysis indicated that no individual SNP exerted a disproportionate influence on the overall estimates (supplementary materials). In the study, reverse MR was employed, which ultimately revealed no significant evidence of causality between NAFLD and the specific plasma lipids identified (**Supplementary Tables S4**, **S5**).

### 3.2 Causal association of plasma metabolome on NAFLD

**Figure 6:**
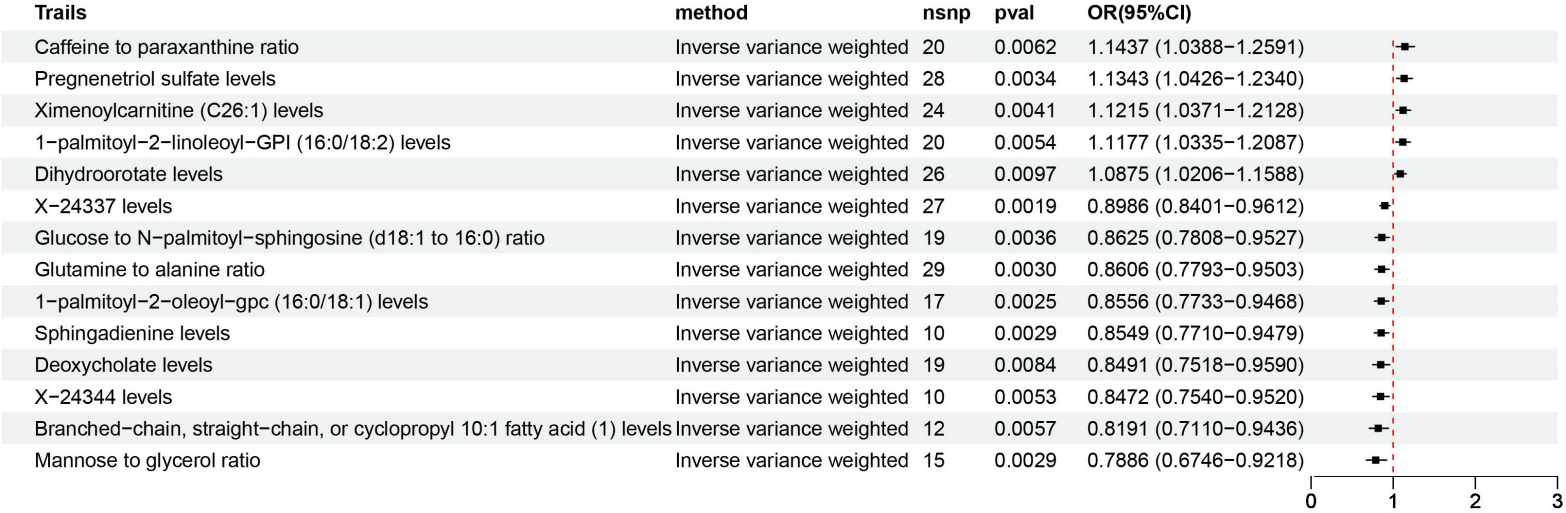
Causal estimates of MR between plasma metabolome and NAFLD. Estimates from the IVW analysis of plasma metabolome on NAFLD. MR, Mendelian randomization; NAFLD, nonalcoholic fatty liver disease; IVW, inverse variance weighted.

**Figure 7:**
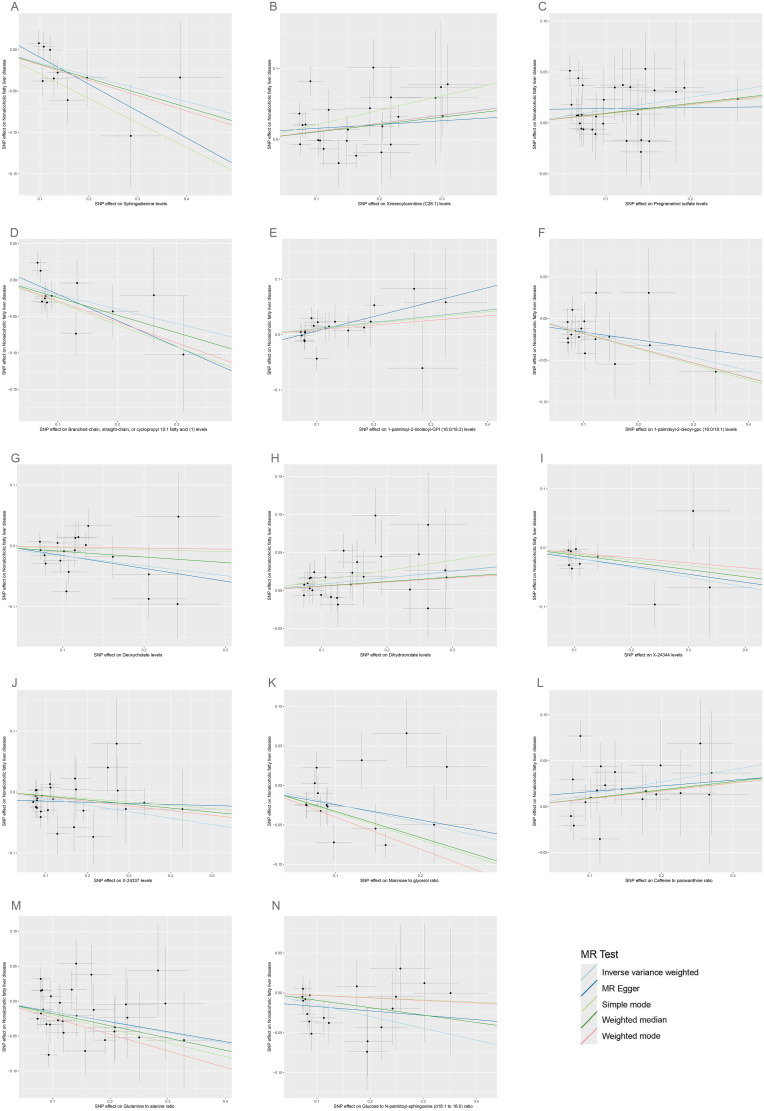
Scatter plots for the causal associations between 14 plasma metabolites on NAFLD. **(A–N)** Each black point represents the SNP effect sizes on the exposure (horizontal axis) and outcome (vertical axis) and is plotted with error bars of SE. The line slopes represent the causal association for each method: inverse variance weighted (light blue line), MR-Egger (blue line), simple mode (light green line), weighted median (green line), and weighted mode (red line). SNP, single-nucleotide polymorphisms; SE, standard error; IVW, inverse variance weighted; SCFA, short-chain fatty acid; BCFA, branched chain fatty acid.

According to IVW estimates, the 14 causal associations between genetically determined plasma metabolites and NAFLD were demonstrated (*P*_IVW_< 0.05; corresponding to 10 metabolites and four metabolite ratios) (**Figures 2B**, **6**, **7**). Additional methods were employed to corroborate these findings (**Supplementary Table S6**). Among them, caffeine-to-paraxanthine ratio (OR: 1.1437/SD increase, 95% CI: 1.0388−1.2591; *p* = 0.0062), pregnenetriol sulfate (OR: 1.1343/SD increase, 95% CI: 1.0426−1.2340; *p* = 0.0034), and ximenoylcarnitine (C26:1) (OR: 1.1215/SD increase, 95% CI: 1.0371−1.2128; *p* = 0.0062) were proven to be the risk factors for NAFLD, while others were protective factors, such as the 1-palmitoyl-2-oleoyl-glycerophosphorylcholine (GPC) (16:0/18:1) levels (OR: 0.8556/SD increase, 95% CI: 0.7733−0.9468; *p* = 0.0025), sphingadienine levels (OR: 0.8549/SD increase, 95% CI: 0.7710−0.9479; *p* = 0.0029), deoxycholate levels (OR: 0.8491/SD increase, 95% CI: 0.7518−0.9590; *p* = 0.0084), branched-chain, straight-chain, or cyclopropyl 10:1 FA (1) levels (OR: 0.8191/SD increase, 95% CI: 0.7110−0.9436; *p* = 0.0057), and glucose-to-N-palmitoyl-sphingosine (d18:1 to 16:0) ratio (OR: 0.8625/SD increase, 95% CI: 0.7808−0.9527; *p* = 0.0036). Scatter plots corroborated the results, demonstrating consistency in their directional trends (**Figure 7**). Except for deoxycholate levels, glutamine-to-alanine ratio and mannose-to-glycerol ratio revealed no heterogeneity and horizontal pleiotropy as tested by both MR-Egger regression and MR-PRESSO (**Supplementary Table S7**). The results remained consistent following the application of the leave-one-out method (**Supplementary Materials**).

**Figure 8:**
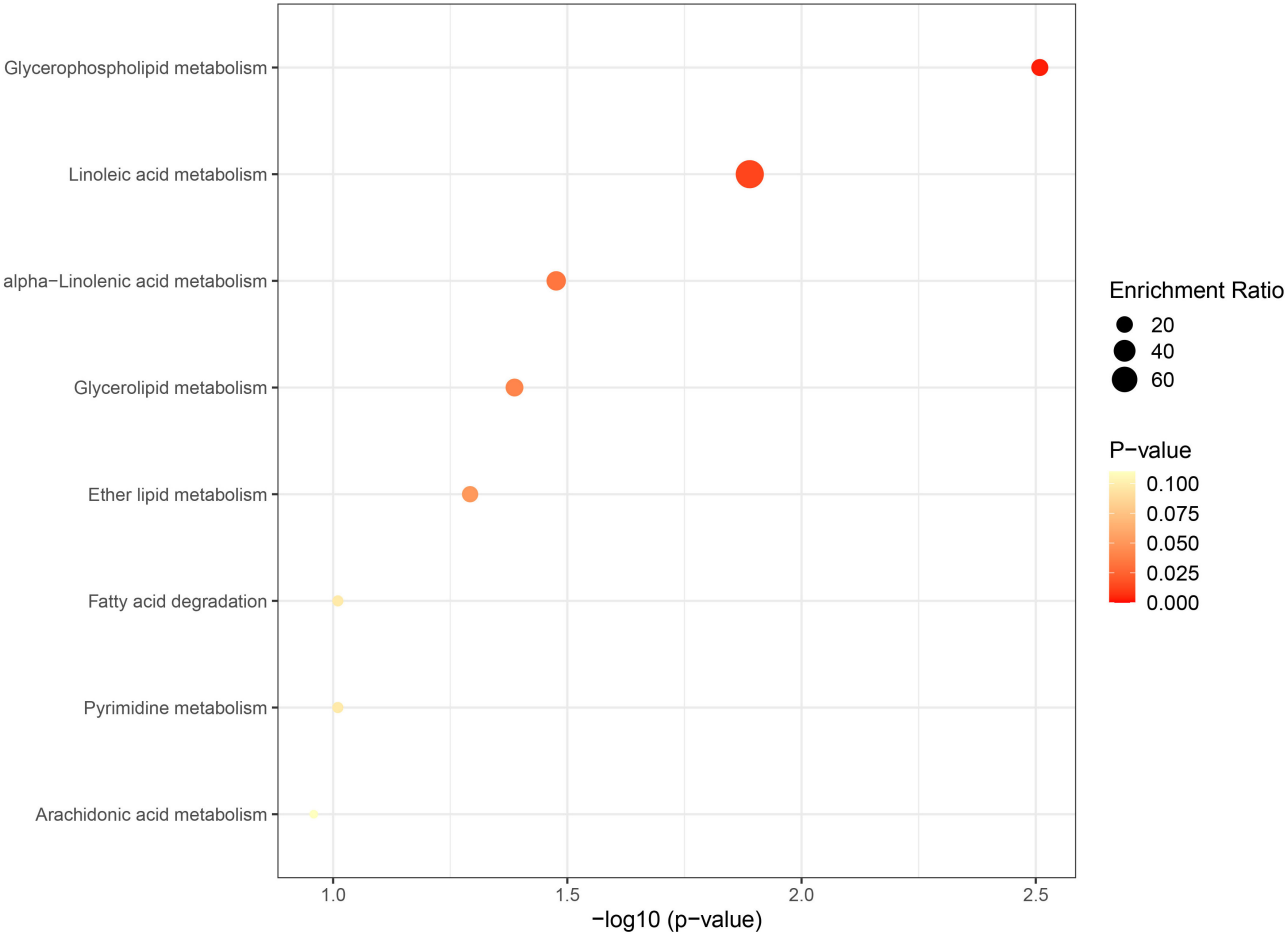
Bubble graph for KEGG pathway enrichment. The horizontal axis represents the pathway impact, and the vertical axis is -log10(p-value). Pathway analyses of identified plasma lipids and metabolites according to the KEGG database. KEGG, Kyoto Encyclopedia of Genes and Genomes.

The metabolic pathway analysis inferred eight related metabolic pathways (**Table 1**; **Figure 8**; **Supplementary Figure S1**). We identified metabolic pathways from plasma lipidomes and metabolomes data, including “glycerophospholipid (GP) metabolism” (*p* = 0.0031; FDR = 0.248), “linoleic acid (LA) metabolism” (*p* = 0.0129; FDR = 0.518), “alpha-linolenic acid (ALA) metabolism” (*p* = 0.0334; FDR = 0.816), “glycerolipid metabolism” (*p* = 0.0410; FDR = 0.816), “ether lipid (EL) metabolism” (*p* = 0.0510; FDR = 0.816), “FA degradation” (*p* = 0.0977; FDR = 1.0), “pyrimidine metabolism” (*p* = 0.0977; FDR = 1.0), and “arachidonic acid metabolism” (*p* = 0.1100; FDR = 1.0).

**Table 1:** Significant metabolic pathways involved in the pathogenesis of NAFLD outlined in the analyses.

Metabolic pathway	Metabolites involved	*P*-value	FDR	Database
Glycerophospholipid metabolism	Phosphatidylcholine, glycerophosphocholine	0.0031	0.248	KEGG
Linoleic acid metabolism	Phosphatidylcholine	0.0129	0.518	KEGG
Alpha-linolenic acid metabolism	Phosphatidylcholine	0.0334	0.816	KEGG
Glycerolipid metabolism	Fatty acid	0.0410	0.816	KEGG
Ether lipid metabolism	Glycerophosphocholine	0.0510	0.816	KEGG
Fatty acid degradation	Fatty acid	0.0977	1	KEGG
Pyrimidine metabolism	Dihydroorotic acid	0.0977	1	KEGG
Arachidonic acid metabolism	Phosphatidylcholine	0.1100	1	KEGG

### 3.3 Causal association of plasma lipidome on plasma metabolomes

The IVW method revealed six causal associations between plasma lipidomes and plasma metabolomes (PIVW< 0.05, corresponding to two plasma lipids and five plasma metabolites) (**Supplementary Table S8**). Among the possible mediators impacting NAFLD, TAG (50:4) levels were related to high levels of ximenoylcarnitine (C26:1) (OR: 1.1350/SD increase, 95% CI: 1.0552−1.2209; *p* = 0.0007) and 1-palmitoyl-2-oleoyl-GPC (16:0/18:1) (OR: 1.1031/SD increase, 95% CI: 1.0097−1.2052; *p* = 0.0297). TAG (52:5) levels were related to high levels of ximenoylcarnitine (C26:1) (OR: 1.1951/SD increase, 95% CI: 1.1161−1.2797; *p* = 3.28E-07) and caffeine-to-paraxanthine ratio (OR: 1.1035/SD increase, 95% CI: 1.0034−1.2135; *p* = 0.0424). Genetically predicted TAG (52:5) levels were related to low levels of pregnenetriol sulfate (OR: 0.9219/SD increase, 95% CI: 0.8586−0.9900; *p* = 0.0252) and glucose-to-N-palmitoyl-sphingosine (d18:1 to 16:0) ratio (OR: 0.8969/SD increase, 95% CI: 0.8114−0.9913; *p* = 0.0331). Horizontal pleiotropy was not detected in the MR analysis when investigating the associations between plasma lipidomes and plasma metabolomes (**Supplementary Table S9**).

### 3.4 Mediation analysis results

To investigate the potential mechanism driving the onset and progression of NAFLD, a mediation analysis was performed to elucidate the causal pathway from plasma lipidomes to NAFLD mediated by plasma metabolomes. This analysis specifically examined previously identified plasma lipids and metabolites associated with NAFLD in the two-sample MR.

Utilizing a two-step MR analysis, we initially assessed the potential causal association between plasma lipids and identified metabolites and subsequently screen the metabolites to examine the causal relationship with NAFLD after correcting for the plasma lipids. Following statistical adjustments, the number of causal correlations between plasma lipids and NAFLD decreased from 14 to five.

In summary, we identified six mediation relationships, two of which exhibit statistical significance and four show potential implications (**Figure 3B**; **Table 2**; **Supplementary Figure S2**; **Supplementary Tables S8**-**S13**). The mediation analysis showed that ximenoylcarnitine (C26:1) levels exhibit significant positive mediation effects (beta = 0.0145, 95% CI: 0.0017–0.0274, *P* = 0.0271) on TAG (50:4) levels and NAFLD with 9.92% (95% CI: 1.12–18.7) proportion that no horizontal pleiotropic pathway existed in the MR analysis as well as beta = 0.0204, 95% CI: 0.0053–0.0356, *P* = 0.0081 on TAG (52:5) levels and NAFLD with 13.5% (95% CI: 3.5–23.5) proportion. The pathway from TAG (50:4) levels to NAFLD can be mediated by 1-palmitoyl-2-oleoyl-GPC (16:0/18:1) levels with a negative mediation effect of -0.0153. In addition, the mediated effects of TAG (52:5) levels on NAFLD via pregnenetriol sulfate levels, caffeine-to-paraxanthine ratio, and glucose-to N-palmitoyl-sphingosine (d18:1 to 16:0) ratio were -0.0102, 0.0132, and 0.0161, respectively. The mediation proportion of the TAG (52:5) caffeine to paraxanthine ratio was found to be 8.72%. Consistent directions for the total, indirect, and direct effect are the premise to achieve mediation proportions.

**Table 2:** Mediation effect of plasma lipidome on NAFLD via plasma metabolites.

Exposure	Mediator	Outcome	Total effect	Direct effect	Mediation effect (95% CI)	*P*-value	Mediation proportion (95% CI)
TAG (52:5) levels	Ximenoylcarnitine (C26:1) levels	NAFLD	0.1515	0.1311	**0.0204** (**0.0053**, **0.0356**)	**0.0081**	**13.50%** (**3.50%**, **23.50%**)
TAG (50:4) levels	Ximenoylcarnitine (C26:1) levels	NAFLD	0.1464	0.1319	**0.0145** (**0.0017**, **0.0274**)	**0.0271**	**9.92%** (**1.12%**, **18.70%**)
TAG (52:5) levels	Glucose to N-palmitoyl-sphingosine (d18:1 to 16:0) ratio	NAFLD	0.1515	0.1354	0.0161 (-0.00221, 0.0344)	0.0848	–
TAG (50:4) levels	1-Palmitoyl-2-oleoyl-gpc (16:0/18:1) levels	NAFLD	0.1464	0.1618	-0.0153 (-0.0333, 0.00271)	0.096	–
TAG (52:5) levels	Pregnenetriol sulfate levels	NAFLD	0.1515	0.1617	-0.0102 (-0.0223, 0.00185)	0.0969	–
TAG (52:5) levels	Caffeine to paraxanthine ratio	NAFLD	0.1515	0.1383	0.0132 (-0.00273, 0.0292)	0.1042	8.72%

# When the 95% Cl of the mediation effect spans 0, the 95% Cl for mediation proportion is not calculated, as the direction of the upper or lower limit of the mediation effect is opposite to the total effect. When the directions of beta 1, beta 2 and beta all are inconsistent, the mediation proportion is not computed. Bold formating indicates that the P-value is less than 0.05. NAFLD, nonalcoholic fatty liver disease; TAG, triacylglycerol; CI, confidence interval.

The sensitivity analysis provided further support for the MR results by effectively excluding the presence of horizontal pleiotropy. Scatter and funnel plots were employed to visually reveal potential biases and data robustness, improving analysis credibility (**Supplementary Figures S3**, **S4**). Furthermore, the leave-one-out analysis presented proof of backing the consistent causality observed in the two-sample MR study among exposure, mediator, and outcome (**Supplementary Figure S5**).

## 4 Discussion

Currently, omics technologies, especially lipidomics and metabolomics, have been utilized in NAFLD research to identify key biomarkers, investigate metabolic processes, and reveal metabolic mechanisms. Nevertheless, the existing evidence is constrained to the examination of individual omics. We first employed genetic variation to conduct natural experiments in order to investigate novel metabolic pathways by integrating lipidome and metabolome analyses. Genetic variants are employed in MR analysis as IVs to estimate causal effects with reduced confounding. In this research, MR was utilized to comprehensively assess the causality between plasma lipidomes, metabolomes, and NAFLD as well as pinpoint five plasma lipids and 14 metabolites as potential causal factors for NAFLD. The pathway analysis revealed eight metabolic pathways related to NAFLD, highlighting the significance of GP, LA, ALA, and glycerolipid metabolism, based on the known compounds in the KEGG. Additionally, we identified six mediation relationships, with five metabolites serving as mediators in the causal pathway from plasma lipids to NAFLD.

Consistent with prior research, our study identified TAG as a primary factor contributing to NAFLD (6, 26). The TAG levels in the liver and plasma increased significantly with the advancement of NAFLD, indicating a link between hepatic TAG buildup and the severity of NAFLD (26). These particular TAGs exhibit longer carbon chains and fewer double bonds (27). Our study findings, which encompassed the levels of TAG (52:5, 50:4, 46:1, and 51:2) aligned well with these characteristics. Alternatively, our findings indicated that a high level of PC (18:1/0:0) may be useful for the prevention of NAFLD. Previous studies have not clearly reported PC (18:1/0:0), while several studies show low levels of certain PC in NAFLD patients (28). Research on metabolic syndrome in animals proposed that increased adipocyte turnover results in decreased PC levels (29). It is hypothesized that a mutation in the Ccna2 gene promoter in obese mice may lead to a heightened mitotic activity in adipose tissue (29). The absence of PC synthesis at the cell membrane causes TAG accumulation in hepatocytes by inhibiting very-low-density lipoprotein secretion (30). Observational studies suggest that administering oral PC can enhance liver enzymes and histopathological changes in clinical and animal trials (28, 31). Different structural forms of PC have been demonstrated to have divergent effects, necessitating further research to investigate the molecular mechanisms and clinical implications of our MR analysis results (32). The pathway analysis suggests that PC is part of the GP, LA, ALA, and arachidonic acid metabolism pathway. GPs are the primary phospholipids found in mammalian cell membranes, such as PC and phosphatidylethanolamine (PE). A decrease of PC/PE was observed as NAFLD advances (33). A combined transcriptomic and metabolomic analysis showed that *Smilax china* L. saponins regulates lipid accumulation by affecting GP metabolism, potentially improving NAFLD (34). LA is a crucial polyunsaturated FA that supports metabolism. Excessive intake may cause NAFLD by disrupting omega-6 and omega-3 balance. LA is transformed into arachidonic acid that has been shown to be pro-inflammatory (35). 17β-estradiol was reported to help the regulation of LA metabolism to prevent postmenopausal NAFLD (36). ALA, a type of omega-3 FA, can be converted into eicosapentaenoic acid and docosahexaenoic acid, which are hypothesized to improve NAFLD by ameliorating lipid accumulation, controlling inflammation and attenuating oxidative stress (37). The KEGG signaling pathway analysis also showed the arachidonic acid metabolism, which is a lipid metabolism-related pathway. The metabolic pathways analyzed were found in agreement with the reported literature (38).

Five plasma metabolomes are involved in mediating the causal relationships between plasma lipids and NAFLD. Ximenoylcarnitine (C26:1) levels were investigated as an excellent mediator in our findings, mediating two pathways linking plasma lipidome and NAFLD with notable statistical significance. The mediation ratio of the ximenoylcarnitine (C26:1) level order TAG (50:4) and TAG (52:5) level to the NAFLD was 9.92% and 13.5%, respectively. The other metabolomes show potential mediating implications, namely, caffeine-to-paraxanthine ratio, pregnenetriol sulfate levels, 1-palmitoy1-2-oleoyl-GPC (16:0/18:1) levels, and glucose to N-palmitoyl-sphingosine (d18:1 to 16:0) ratio. In a case–control study of patients with diabetic retinopathy, the significant correlations of ximenoylcarnitine (C26:1) were reported with many GP and acylcarnitine species, reflecting alterations in lipid homeostasis (39). Indeed both acylcarnitines and GPs demonstrate a positive correlation between their accumulation and NAFLD progression (40). To ensure the therapeutic effect and safety of medication, individualized medicine in patients with NAFLD is necessary. Caffeine is metabolized to paraxanthine primarily by CYP1A2, and the caffeine-to-paraxanthine ratio measured in plasma is used to assess CYP1A2 enzyme activity (41). Studies have found that caffeine/paraxanthine levels are doubled in NAFLD patients, caused by a decreased CYP1A2 activity (42). Evidence suggests that caffeine consumption may elevate the risk of developing NAFLD. Therefore, more attention should be paid to avoid the potential risk. Pregnenetriol sulfate, a steroid hormone derivative of 17-hydroxy-pregnenolone, contributes to elevated levels of endogenous steroid hormones, which are implicated in the progression of NAFLD (43, 44). 1-Palmitoyl-2-oleoyl-GPC (16:0/18:1) is a GPC that contains palmitic acid and oleic acid as its long-chain FA constituents. Within the NAFLD patients, GPC levels exhibited a downward trend from non-NASH to NASH to cirrhosis (45). This is consistent with the results obtained by our findings that GPC confers a protective effect against the development of NAFLD. GPCs are involved in EL metabolism pathway, where membrane homeostasis and trafficking process take place. The ratio of glucose to N-palmitoyl-sphingosine (d18:1 to 16:0) is thought to reflect glucose homeostasis, which is advantageous for individuals with NAFLD (46). Glutamine-to-alanine ratio, which reflects alterations in amino acid metabolism, exhibits potential as negative correlation indicators of NAFLD (6).

We report for the first time that sphingadienine and cyclopropyl FA serve as effective protective agents, contributing to the inhibition in the advancement of NAFLD. Further research will be necessary to confirm the notions. The administration of short-chain fatty acids ameliorated NASH by demonstrating significant efficacy in reducing hepatic steatosis and inflammation through the enhancement of AMP-activated protein kinase activation and the mitigation of proinflammatory reactions in macrophages, respectively (47). Similarly, a decrease in branched chain fatty acids was observed in the livers of NAFLD patients, potentially attributable to the downregulation of biosynthetic pathways or the diminished mitochondrial function in adipose tissue (26).

Deoxycholate is the sodium salt form of deoxycholic acid, the increased level of which serves as the endogenous antagonists of farnesol X receptor, thereby promoting NAFLD (48). This verdict is contrary to the finding reached by us that deoxycholate acts as a protective factor for NAFLD. It is hypothesized that an increase in deoxycholate concentration may lead to the selection of gut microbiota that facilitate the proliferation of beneficial bacteria.

The glycophosphatidylinositol (GPI) molecule 1-palmitoyl-2-linoleoyl-GPI (16:0/18:2) contains palmitic acid and linoleic acid linked to the first and second carbons of glycerol, respectively. It is hypothesized that increased hepatic glycosylphosphatidylinositol-specific phospholipase D in NAFLD cleaves GPI anchor to be released into the plasma, leading to liver diacylglycerol accumulation and the onset of insulin resistance (49). Dihydroorotate (DHO) serves as an intermediary in pyrimidine metabolism, with DHO dehydrogenase functioning as an enzyme catalyzing the oxidation of DHO to uridine monophosphate. The inhibition of DHO dehydrogenase activity leads to the buildup of DHO, resulting in a depletion of hepatic pyrimidines, thereby exacerbating the progression of NAFLD (50).

This research represents the inaugural utilization of a comprehensive MR analysis to investigate the causal link between plasma lipidome, plasma metabolome, and NAFLD. These lipids connected to metabolites, suggesting that these lipid–metabolite correlations associate with NAFLD. Furthermore, a pathway from lipidome to NAFLD was elucidated through a mediation analysis of plasma metabolites. Various sensitivity analyses were employed to enhance the reliability of our results. However, some limitations existed in this study. Firstly, our MR analysis only included individuals of European descent in the GWAS, thereby restricting the applicability of the results to diverse populations. Secondly, residual and unmeasured confounders may persist, as the study was unable to ascertain the influence of demographic stratification and other potential confounders on its findings. Additional experimental and clinical studies are required to validate this result despite the effectiveness of the MR method in assessing the causal relationship. Thirdly, due to the data limitations of KEGG, our pathway analysis reveals that up to two metabolites were enriched within a single pathway without high statistical significance after FDR correction. Lastly, the data for the plasma lipidome and metabolome could not offer direct insights into alterations within the liver. Nevertheless, there is no gainsaying the fact that our study reports several novel and valuable observations regarding the systemic metabolic status in NAFLD.

## 5 Conclusion

In summary, this MR analysis supports the causal impacts of plasma lipidomes and metabolomes on NAFLD. Plasma lipidomes and metabolomes provide insights into the potential metabolic pathways linked to NAFLD. Furthermore, it was concluded that plasma metabolites act as causal mediators in the pathway connecting plasma lipids and NAFLD. Indeed the impact of plasma lipidomes on NAFLD is not solely mediated through plasma metabolomes, thereby necessitating further investigation into additional potential mediators. The identified plasma lipids and metabolites can serve as potential plasma biomarkers for the diagnosis and treatment of NAFLD as well as enhance our understanding of the underlying mechanisms of NAFLD.

## Data Availability

The original contributions presented in the study are included in the article/**Supplementary Material**. Further inquiries can be directed to the corresponding author.
