# Supplementary material for: Role of plasma metabolome in mediating the effect of plasma lipidome on NAFLD: a Mendelian randomization study
Source: Front Endocrinol (Lausanne). 2025 Jan 23;15:1436827. doi: 10.3389/fendo.2024.1436827 (PMC11798786; doi:10.3389/fendo.2024.1436827)
Supplement: Supplementary file 1 [file DataSheet1.pdf]

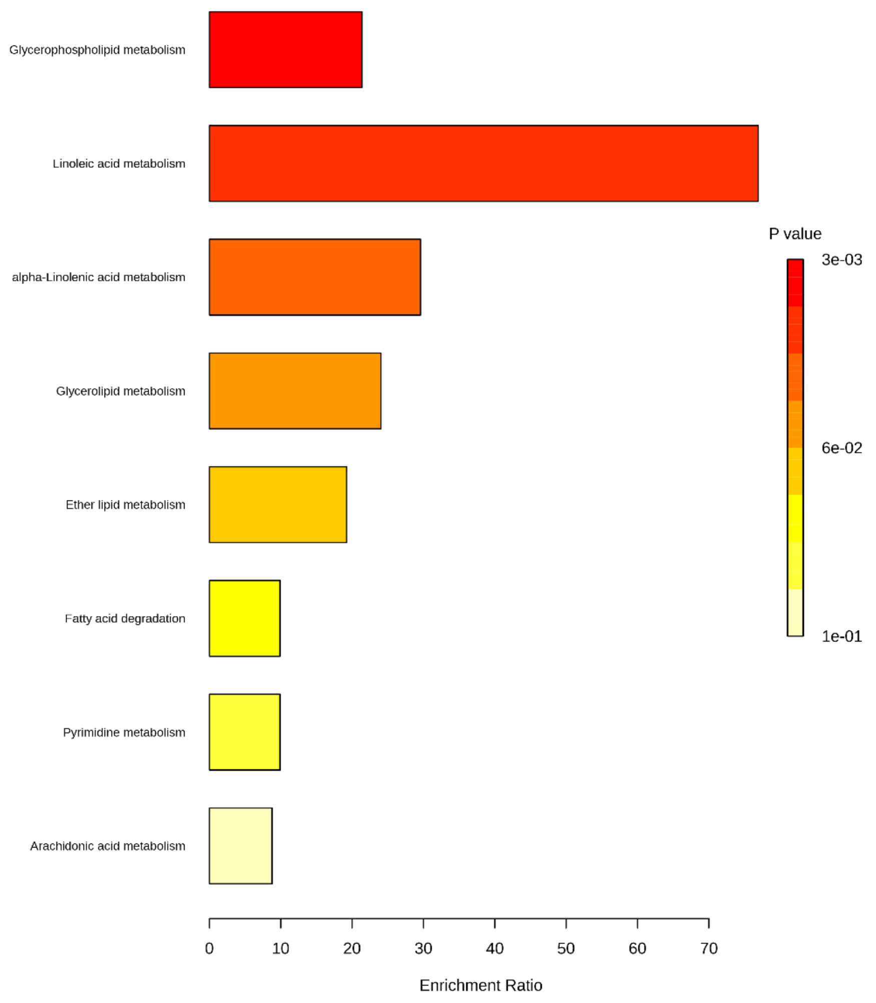

Figure S1 Enriched significant metabolic pathways of NAFLD-associated metabolites.

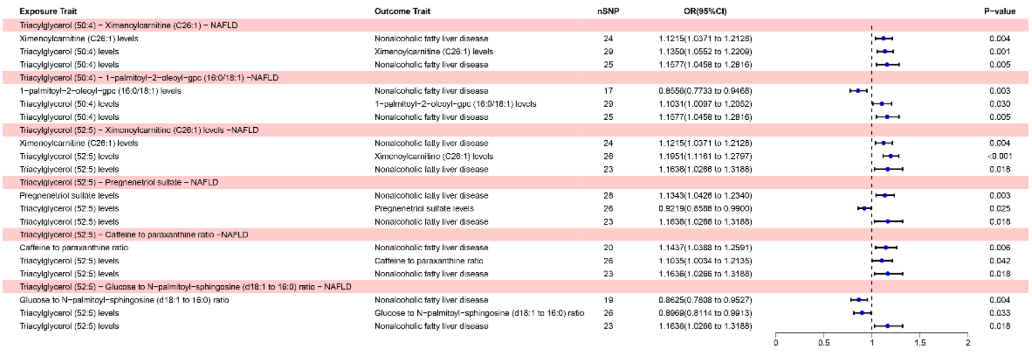

Figure S2 Forest plots for MR analyses of plasma metabolites on NAFLD after adjusting for plasma lipids.

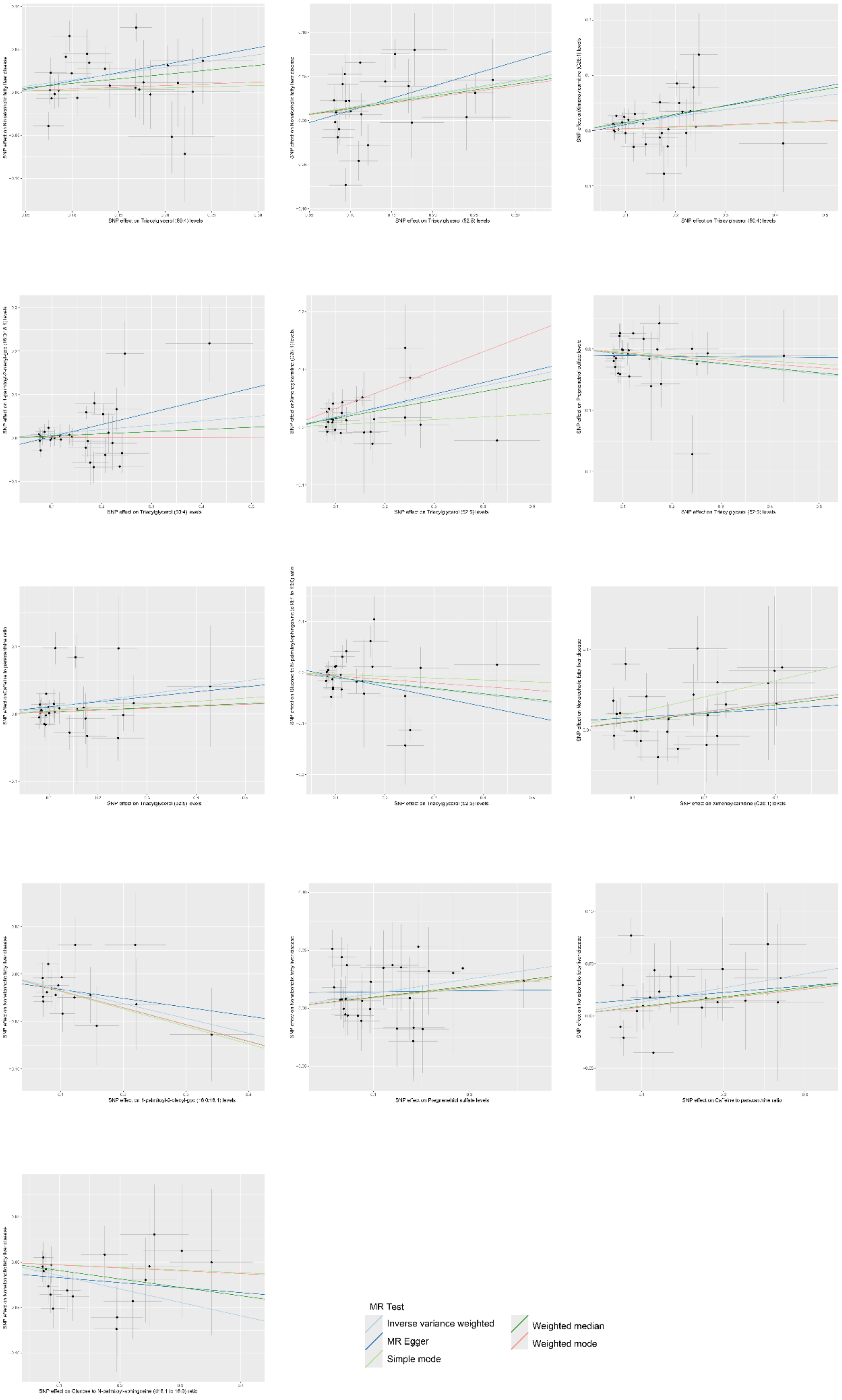
MR Test
Inverse variance weighted
MR Egger
Simple mode
Weighted median
Weighted mode

Figure S3 Scatter plots for MR analyses of plasma metabolites on NAFLD after adjusting for plasma lipids.

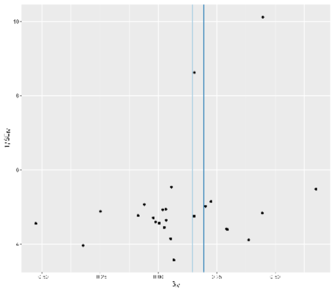

Funnel plot for [illegible] levels on Nonalcoholic fatty liver disease

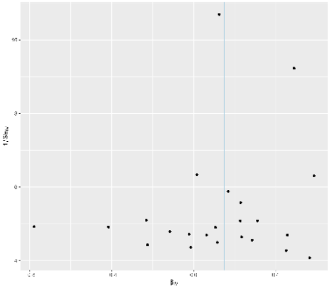

Funnel plot for diacylglycerol [illegible] levels on Nonalcoholic fatty liver disease

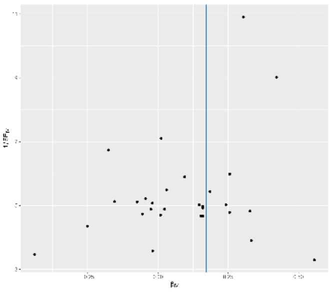

Funnel plot for [illegible]

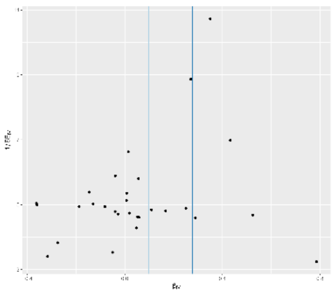

Funnel plot for [illegible] levels

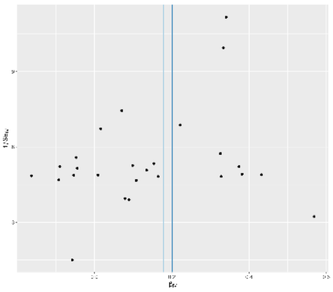

Funnel plot for [illegible] levels

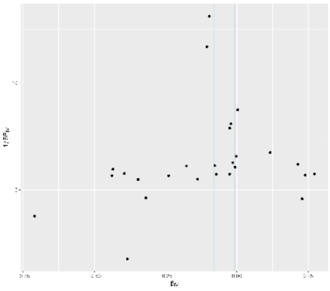

Funnel plot for [illegible]

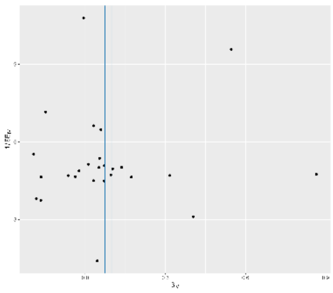

Funnel plot for [illegible] levels on Caffeine to paraxanthine ratio

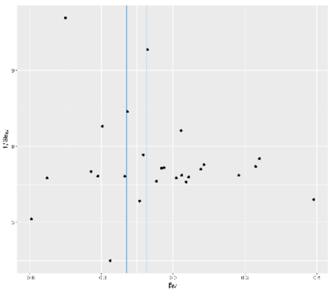

Funnel plot for [illegible] levels on Glucose to N-palmitoyl-sphingosine [illegible] ratio

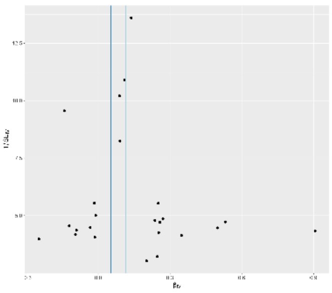

Funnel plot for [illegible] levels on Nonalcoholic fatty liver disease

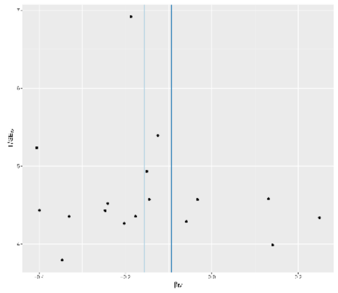

Funnel plot for [illegible] levels on Nonalcoholic fatty liver disease

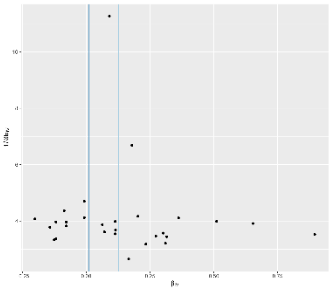

Funnel plot for Pregnenolone sulfate levels on Nonalcoholic fatty liver disease

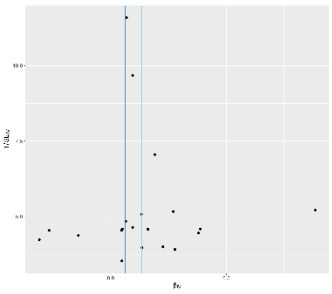

Funnel plot for Caffeine to paraxanthine ratio on Nonalcoholic fatty liver disease

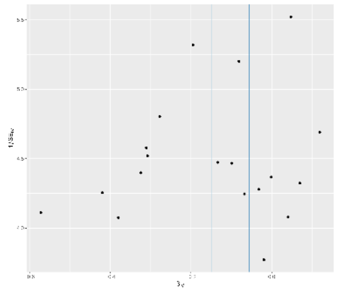

Funnel plot for [illegible] on Nonalcoholic fatty liver disease

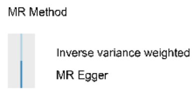

Figure S4 Funnel plots for MR analyses of plasma metabolites on NAFLD after adjusting for plasma lipids.

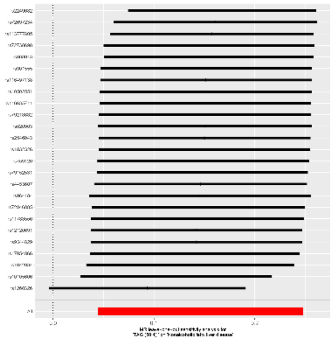
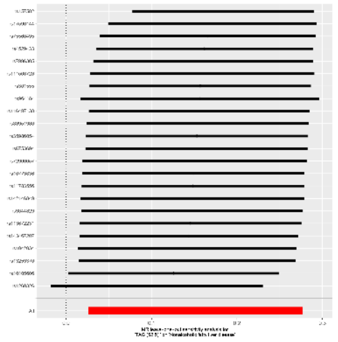
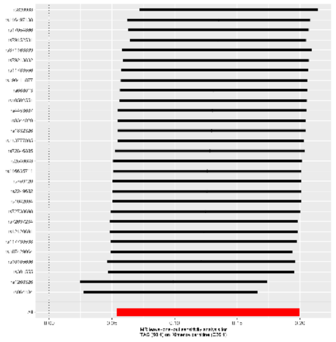
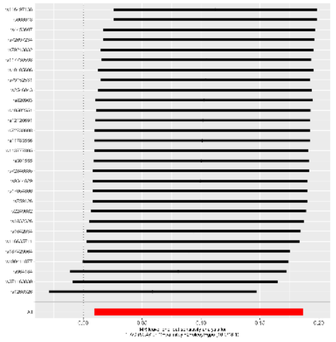
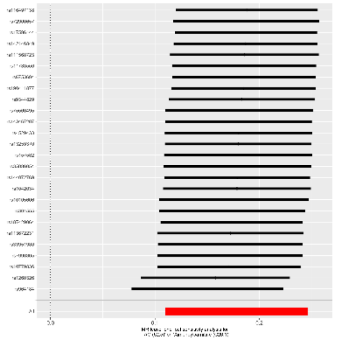
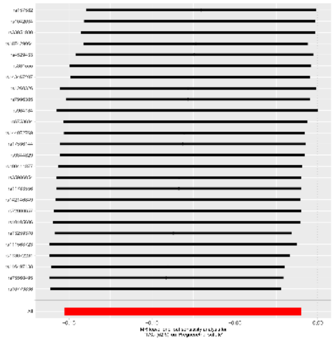
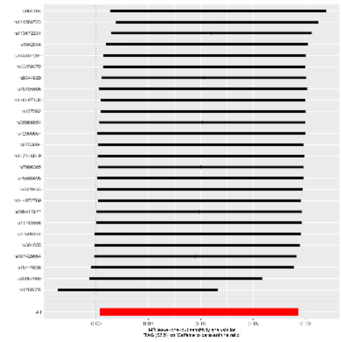
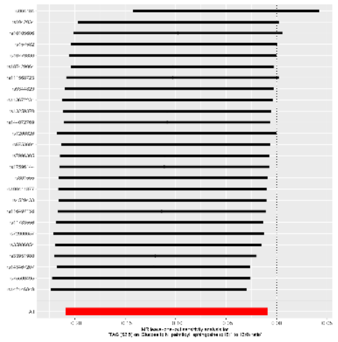
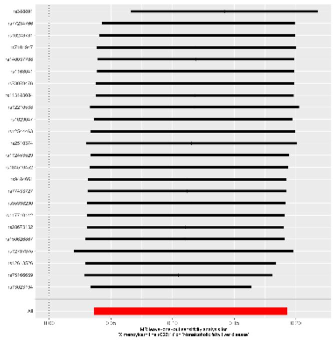
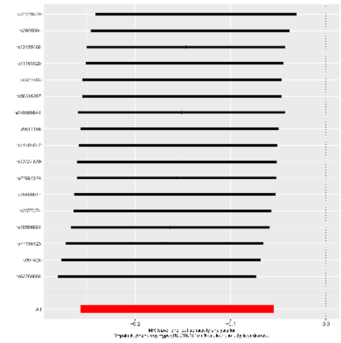
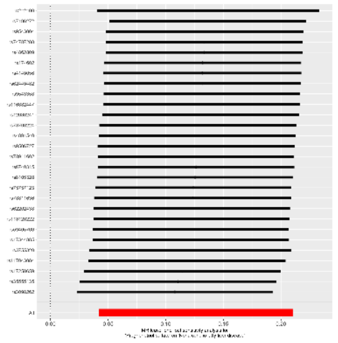
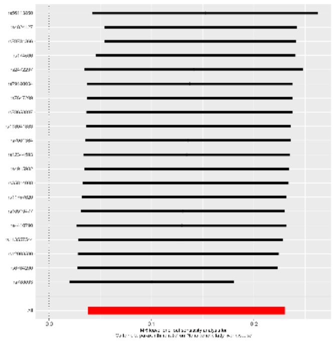
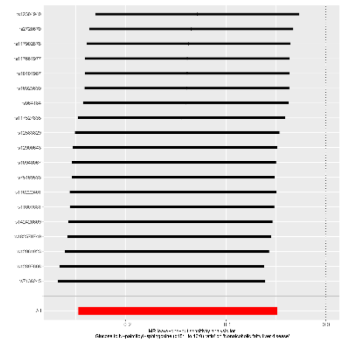

Figure S5 Leave-one-out plots for two-sample MR analysis of plasma lipidome on NAFLD, plasma lipidome on identified plasma metabolome, and identified plasma metabolome on NAFLD. The dark dots indicate effect measures from IVW MR analysis excluding the index SNPs. The red lines indicate the effect size from pooled analysis including all SNPs according to the IVW MR method. NAFLD, nonalcoholic fatty liver disease; TAG, triacylglycerol; SNP, single-nucleotide polymorphisms; IVW, inverse variance weighted; MR, Mendelian randomization.
